# Epidemiological characteristics of common respiratory pathogens in children

**DOI:** 10.1038/s41598-024-65006-3

**Published:** 2024-07-15

**Authors:** Guojian Lv, Limei Shi, Yi Liu, Xuecheng Sun, Kai Mu

**Affiliations:** 1 Medical Genetics, Zibo Maternal and Child Health Hospital, Zibo, 255000 China; 2 Clinical Laboratory of Zibo Hospital of Traditional Chinese Medicine, Zibo, 255000 China

**Keywords:** Children respiratory tract infection, Multiple PCR detection of respiratory tract, Pathogens, Positive detection rates, Health occupations, Medical research

## Abstract

Children's respiratory tract infection is a common disease affecting children's health, our purpose is to describe the epidemiological characteristics of common pathogens of children's respiratory tract infection in central Shandong, China, and compare them with those in other parts of world, so as to summarize the rules of children's respiratory tract infection in central Shandong, and provide scientific basis for health departments to prevent and treat local children's respiratory tract infection. Sputum, tracheal aspirate, alveolar lavage fluid and other samples of 4804 children admitted to wards of Zibo Maternal and Child Health Hospital for treatment of respiratory tract infection from June 2019 to December 2022 were collected, and 12 common respiratory tract pathogens were detected by PCR capillary electrophoresis fragment analysis, two bacteria (*Streptococcus pneumoniae, Haemophilus influenzae*), two atypical pathogens (*Mycoplasma Pneumoniae*, *Chlamydia Pneumoniae*) and eight viruses (*Human rhinovirus*, *Respiratory Syncytial Virus*, *Influenza A Virus*, *Parainfluenza Virus*, *Human metapneumovirus*, *Human boca virus*, *Human coronavirus*, *Influenza B virus*) were included, the positive detection rate of single pathogen, the proportion of each type of respiratory tract mixed infection and the positive detection rate of single pathogen in different ages and seasons were analyzed statistically. (1)Among 4804 children with respiratory tract infection, the total positive rate was 77.87% (3741/4804), the positive rate of single pathogen was 43.40% (1656/4804), *Streptococcus pneumoniae*, *Rhinovirus* and *Respiratory syncytial virus* were the highest, there were 2085 cases of mixed infection with two or more pathogens, the positive rate was 43.40%. (2) The positive rates of infection in infant group (0–1 years old), infant group (1–3 years old), preschool group and school age group (3 years old-) were roughly the same, the infection rates of *Streptococcus pneumoniae*, *Respiratory syncytial virus* and *Parainfluenza virus* in infant group, *Rhinovirus* in infant group, *Influenza A virus*, *Chlamydia pneumoniae*, *Mycoplasma pneumoniae* and *Haemophilus influenzae* in school age group were higher than those in other groups, the difference was statistically significant (*P* < 0.05). (3) The positive detection rates of spring, summer, autumn and winter groups were 43.58%, 38.64%, 33.73% and 29.27%, respectively, the positive rates of *Streptococcus pneumoniae* and *Haemophilus influenzae* in spring group, *Mycoplasma pneumoniae* in summer group, *Rhinovirus*, *Respiratory syncytial virus* and *Influenza A virus* in autumn group, *Chlamydia pneumoniae*, *Boca virus* and *Influenza B virus* in winter group were higher than those in other seasons, and the differences were statistically significant (*P* < 0.05). The pathogen detection rate of children varies with age and season, and the prevention and treatment of a certain respiratory pathogen infection must be combined with its raging season and age rule.

## Introduction

A respiratory tract infection is a type of infectious illness caused by a range of harmful microorganisms such as viruses, bacteria, fungi, mycoplasma, chlamydia, Rickettsia, parasites, and others^1^. Children's respiratory tract infection is a global problem affecting children's health, especially in China, due to the differences in climate, environment and living habits in different regions, the positive detection rate of various respiratory tract pathogens is different, which brings great difficulties to the prevention and treatment of children's respiratory tract infection. At present, the lack of epidemiological data on pathogens that cause respiratory infections in children in the region has resulted in poor health sector performance in the prevention of respiratory infections in children, and epidemiological data from pathogens that cause respiratory infections in children are needed to determine the season and age groups susceptible to pathogens, and to develop timely strategies for vaccine use. The purpose of this study was to understand the epidemiological characteristics of pathogenic bacteria in different ages and seasons in central Shandong Province, compare the data of respiratory tract infection in children in this region with other infection data around the world, and analyze the rule of respiratory tract infection caused by various pathogens in this region, so as to provide scientific basis for higher health departments to carry out the prevention and control of children respiratory tract infection and vaccine prevention.

## Materials and methods

*Statement 1* The experimental protocol of this study has been reviewed by the Ethics Committee of Zibo Maternal and Child Health Hospital, and the experimental protocol has been sent to the guardians of each sick child in the form of informed consent, and each guardian has signed and agreed.

*Statement 2* Confirm that all methods of this experimental protocol are performed in accordance with relevant guidelines and regulations.

### Research object

A total of 4804 children who came to our hospital for treatment of respiratory tract infections from June 2019 to December 2022 were selected as the study objects for PCR detection to detect the infection of respiratory tract viruses, bacteria, mycoplasma, chlamydia and other pathogens. The detection of pathogens in different age groups was divided into infant group (0–1 years old, n = 1955), Toddler group (1–3 years old, n = 1215), preschool group and school age group (3 years old-, n = 1634), the detection of respiratory pathogens in different seasons was divided into spring group (n = 569), summer group (n = 885), autumn group (n = 1862) and winter group (n = 1488). Inclusion criteria: ① Children with respiratory tract infection were selected according to the 8th edition of Zhufutang Practical Pediatrics^2^ and Internal Medicine^3^; ② Children with stable disease development; ③ With the consent of the guardian, children can actively participate in the treatment.

### PCR capillary electrophoresis fragment analysis of children's respiratory tract

PCR capillary electrophoresis fragment analysis was utilized to identify 12 common respiratory pathogens, which included *Streptococcus pneumoniae* (*S. pneumoniae*), *Haemophilus influenzae* (H. influenzae), *Human rhinovirus* (HRV), *Respiratory Syncytial Virus* (RSV), *Influenza A Virus* (FLUA), *Chlamydia Pneumoniae* (C. pneumoniae), *Mycoplasma Pneumoniae* (M. pneumoniae), *Parainfluenza Virus* (PIV), *Human metapneumovirus* (HPMV), *Human boca virus* (HBOV), *Human coronavirus* (HCOV), and *Influenza B virus* (FLUB).

Primers sequence of each pathogen: *S. pneumoniae*: Reagent company did not provide; HRV: 5'-TGTGCTTGGTTGTGATTCCTCC-3'and5'-GGACACCCAAAGTAGTCGGTC-3'; RSV: 5'-AGCCATTGTGTCATGCTATGG-3'and5'-TAGGCTCTGGTTAATCTTCTCATTGA-3'; FLUA:5'-TCATGGAATGGCTAAAGA-3'and5'-TACGCTGCAGTCCTCGCT-3'; C. pneumoniae: 5'-TACCCGTTGGATTTGAGCGT-3'and5'-TCTAGAAAAATAGTTTTAGATGCCGATTC-3'; M. pneumoniae:5'-GCGACGGGACTCACCGTAG-3'and5'-CGCTGTTGTCGCGCACTAA-3'; H. influenzae:Reagent company did not provide; PIV:5'- TGACAGTATCCTCCGTGAACGAGA-3'and5'-TGCATGACTTCTCTATTAATTGTGTGA-3'; HPMV: 5'-TGGTATTGTAAAAATGCAGGATCCACT-3'and5'-GTGCAACCATGCTGATAGGGTG-3'; HBOV: 5'-GCAGCTCTTCTACAGTGGTGT-3'and5'-GAATGAGTAGGACAAAGGACCCCAA-3'; HCOV: 5'-GTGATGCAACTACTGCTTTTGCTA-3'and5'-AATCAGAGTTATAACASACAACMCCATCA-3'; FLUB:5'-TGGAGTGAGACGAGAAATGCAGAT-3'and5'-GAATTTCCCATGGAGCTCTGCTTTA-3'; huRNA: 5'-TGGAGGCTATCCAGCGTACT-3'and5'-CAATTCTCTCTCCATTCTTCAGT-3'; huDNA: 5'-CCTGCACCACCAACTGCTTA-3'and5'-ACATCACCCCTCTACCTCCC-3'.

Genes used as markers for each pathogen are as follows: *S. pneumoniae* (not provided by reagent company), HRV (5' non-coding Region Gene), RSV (fusion protein gene), FLUA (matrix protein 2 and matrix protein 1 genes), C. pneumoniae (16S ribosomal RNA gene), M. pneumoniae (P1 gene for P1 adhesin protein), H. influenzae (not provided by reagent company), PIV (hemagglutinin-neuraminidase gene), HPMV (fusion protein gene), HBOV (non-structural protein 1 gene), HCOV (RNA-dependent RNA polymerase gene), FLUB (matrix protein 2 and matrix protein 1 genes), huRNA (B2M), and huDNA (GAPDH).

#### Specimen collection

*Throat swab sample* press the patient's tongue backward with tongue depressor, stretch the throat swab into the child's throat to stimulate the cough, stick the coughed sputum on the throat swab, swirl the sample tube fully for 10 s, rinse the cells containing the virus, and send the sample to the nurse assistant within 30 min.

*Sputum sample* Add equal volume of normal saline into the sample tube of sputum collection, thoroughly mix, absorb supernatant for use.

#### Nucleic acid extraction

As per the specifications outlined in the nucleic acid extraction reagent guidelines (Ningbo Helshi Gene Technology, registration number: Zhejiang Yongshibei 20,150,246), the appropriate amount of samples (including positive and negative controls) were taken and combined with 2ul of RT-PCR internal standards. The Shengxiang nucleic acid extraction instrument (Natch32A, Shengxiang Biotechnology Co., LTD., China) was then utilized for the extraction process.

#### RT-PCR (reverse transcription-polymerase chain reaction) amplification

After the RT-PCR system was configured, the samples were placed on ice, the number of samples (including negative control and positive control) were calculated, appropriate amount of respiratory premix and RT-PCR enzyme liquid were added to the centrifuge tube, the mixed liquid of the centrifuge tube was mixed upside down and separated for 10 s, and 15 ul of the mixed liquid was divided into eight tubes for 10 s. The samples were added to the PCR tube and amplified by PCR (GeneAmp PCR System 9700, Applied Biosystems, America) (94°C 30s, 65–60°C 30s, 72°C 60s 6cycle). 94°C 30s, 60 30s, 72°C 60s 29cycle).

#### Detection of fluorescence signal intensity by capillary electrophoresis apparatus

The 1 ul standard was diluted into a 10 ul system and transferred to a genetic analyzer (3500xL Dx Gene Analyzer, Applied Biosystems, America) to obtain the peak height of the standard in each capillary tube.

#### Capillary electrophoresis separation of PCR products

The 1 ul PCR product was diluted into a 10 ul system and transferred to a gene analyzer (3500xL Dx Gene Analyzer, Applied Biosystems, America) to obtain the peak height of the PCR product in each capillary tube.

#### Quality control

The peak pattern using positive control as the template must have 12 pathogen characteristic peaks and 3 internal reference peaks, with a total of 15 peaks, and the peak height of the 15 peaks is higher than the standard peak in the capillary channel, and the absolute difference between the actual fragment length and the reference fragment length is less than 1.5 nt, the peak pattern using negative control as the template must have human DNA internal reference peak and RT-qPCR internal reference peak, and the peak height of these two internal reference peaks is higher than the standard product peak in the capillary channel, human RNA internal reference peak can appear or not appear, and the characteristic peak of pathogens detected by this kit cannot appear, the absolute difference between the actual fragment length and the reference fragment length is less than 1.5 nt.

#### Result determination

*Negative result determination* when there is no pathogen characteristic peak or pathogen characteristic peak is negative, if there is human DNA internal reference peak, human RNA internal reference peak and RT-qPCR internal reference peak and the 3 internal reference peaks are higher than the standard peaks, the detection result is interpreted as negative; If there is no human DNA internal reference peak or human RNA internal reference peak or peak height is lower than the standard product peak, then the detection failure is considered and the sample should be extracted and tested again.

*Positive results* If the peak of the target site of the amplified product is higher than the peak of the capillary standard product, it is judged to be positive.

### Statistical analysis

The data were processed by Graphpad prism software, *χ*^*2*^ test was used for counting data, represented by n(%), *P* < 0.05, the difference of 0.05 was statistically significant.

### Ethical approval

It has passed the review of Ethics Committee of Zibo Maternal and Child Health Hospital Consent for publication.

## Result

### Detection of pathogens in children with respiratory tract infection

**Figure 1:**
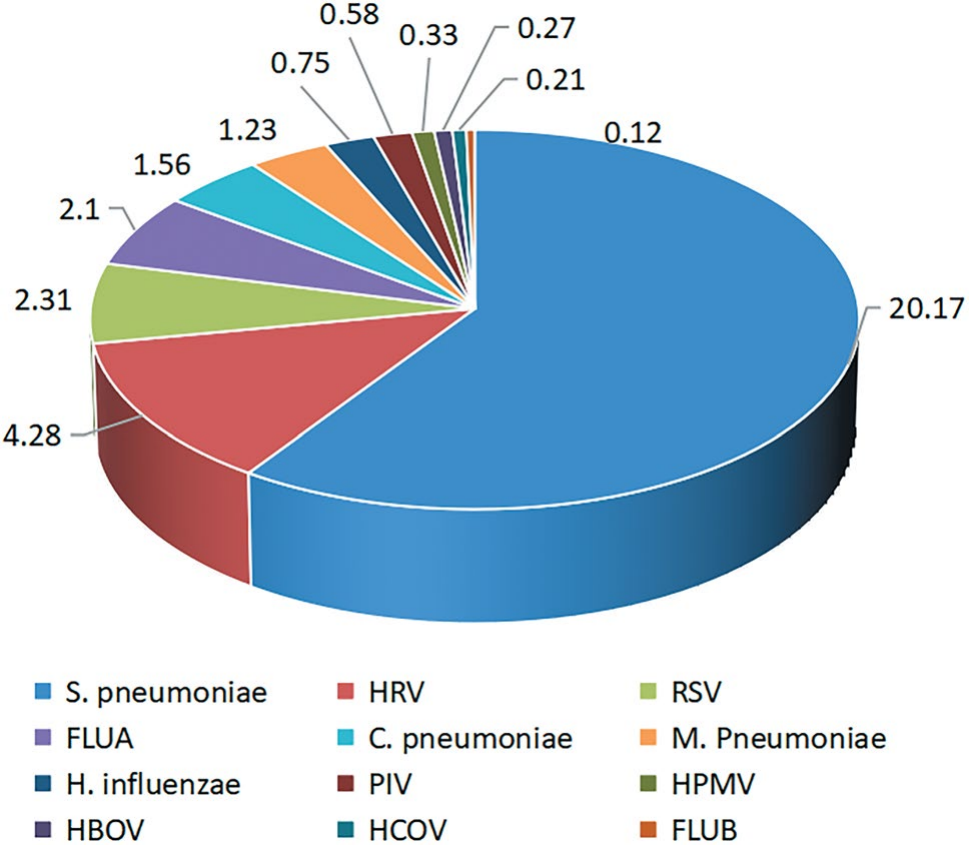
Positive rate and proportion of single pathogen infection in respiratory tract.

The pathogen that causes respiratory infection in children may be bacterial, viral, mycoplasma or chlamydia infection, or a combination of two or more pathogens, therefore, depending on whether the child's lymphocytes are elevated, whether the body temperature exceeds 38.5 °C, whether the child is accompanied by poor mental state, pale face, muscle pain and other symptoms, PCR detection of respiratory pathogens was performed in 4804 children, and the detection range was common bacteria, viruses, mycoplasma, chlamydia, and other pathogens causing respiratory infections. The test results are as follows: 3741 positive samples, the positive rate is 77.87%. The number of single infection was 1656, the positive rate was 34.47%. The higher detection rates were *Streptococcus pneumoniae,* and *Respiratory syncytial virus*, accounting for 20.17%, 4.28% and 2.31%, respectively. The results are shown in Table 1 and Fig. 1.

**Table 1:** Positive rate and proportion of single pathogen infection in respiratory tract.

Pathogen	Positive number (*n*)	Positive rate (%)	Account for (%)
S. pneumoniae	969	20.17	58.51
HRV	205	4.28	12.28
RSV	111	2.31	6.70
FLUA	101	2.10	6.10
C. pneumoniae	75	1.56	4.53
M. Pneumoniae	59	1.23	3.56
H. influenzae	36	0.75	2.17
PIV	28	0.58	1.69
HPMV	16	0.33	0.97
HBOV	13	0.27	0.79
HCOV	10	0.21	0.60
FLUB	6	0.12	0.36
total	1656	34.47	100

The pathogens with high positive rate were Streptococcus pneumoniae, Rhinovirus and Respiratory syncytial virus.

### Analysis on detection of respiratory pathogens mixed infection in children

Common pathogens were detected in 4804 children with respiratory tract infection. The results showed that 2085 children had mixed infection, with a positive rate of 43.40%, there were 43 kinds of mixed infection combinations. The mixed infection combinations with high positive detection rate were *Streptococcus pneumoniae* + *Rhinovirus*, *Streptococcus pneumoniae* + *Haemophilus influenzae*, *Streptococcus pneumoniae* + *Respiratory syncytial virus*, accounting for 12.90%, 10.84% and 8.30%, respectively, other high combinations were *Streptococcus pneumoniae* + *Chlamydia pneumoniae*, *Streptococcus pneumoniae* + *Mycoplasma pneumoniae*, *Streptococcus pneumoniae* + *Rhinovirus* + *Haemophilus influenzae*, etc., the other combinations were not statistically analyzed because they involved more pathogens and the number of cases was too small. The results are shown in Table 2.

**Table 2:** Analysis of mixed infection of respiratory pathogens in children.

Pathogen	Positive number (n)	Account for (%)
S. pneumoniae + HRV	269	12.90
*S. pneumoniae* + H. influenzae	226	10.84
S. pneumoniae + RSV	173	8.30
*S. pneumoniae* + C. pneumoniae	141	6.76
*S. pneumoniae* + M. Pneumoniae	121	5.80
S. pneumoniae + FLUB	72	3.45
*S. pneumoniae* + HRV + H. influenzae	65	3.12
S. pneumoniae + HPMV	58	2.78
*S. pneumoniae* + H. influenzae + RSV	50	2.40
Other combinations	910	43.65
total	2085	100

### Detection and analysis of respiratory single pathogen infection in children of different age groups

**Figure 2:**
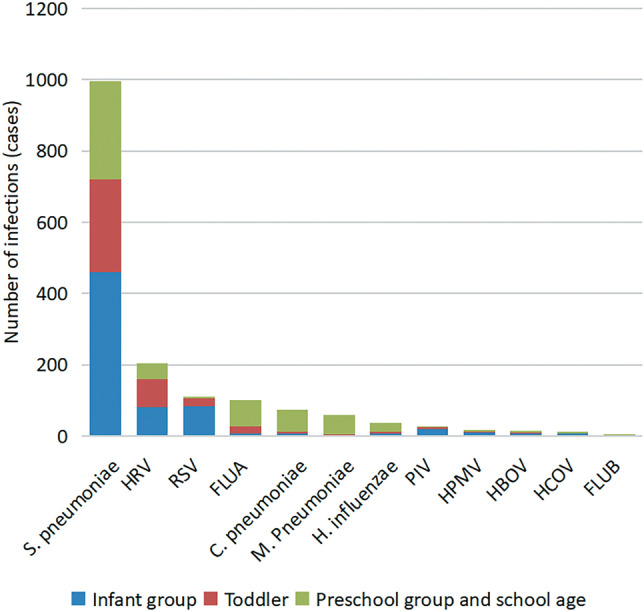
Comparative analysis of respiratory single pathogen infection in children of different age groups.

Common pathogens were detected in 4804 children with respiratory tract infection. The prevalence of respiratory tract single pathogens in children of different age groups was different, the positive rate of respiratory tract single pathogen detection in infant group (0–1 years old) was 35.40% (692/1955), the positive rate of respiratory single pathogen detection was 33.58% (408/1215) in Toddler group (1–3 years old), and 34.03% (556/1634) in the preschool group and the school age group (3 years old -), due to the small number of specimens in the school age group, the preschool group and the school age group were combined into one group for the convenience of analysis. The infection rates of *Streptococcus pneumoniae, Respiratory syncytial virus* and *Parainfluenza virus* in infant group were higher than those in Toddler group, preschool group and school age group, and the difference was statistically significant compared with other age groups (*P* < 0.05); The infection rate of *Rhinovirus* in Toddler group was higher than that in the infant group, preschool group and school age group, and the difference was statistically significant compared with other age groups (*P* < 0.05); The infection rates of *Influenza A virus*, *Chlamydia pneumoniae*, *Mycoplasma pneumoniae* and *Haemophilus influenzae* in preschool and school age groups were higher than those in infant group and Toddler group, and the difference was statistically significant compared with other age groups (*P* < 0.05); There was no significant difference in the infection rates of *Metapneumovirus*, *Boca virus*, *Coronavirus* and *Influenza B virus* (*P* > 0.05). The results are shown in Table 3 and Fig. 2.

**Table 3:** Comparative analysis of respiratory single pathogen infection in children of different age groups.

Pathogen	Infant group (n = 1955)		Toddler group (n = 1215)		Preschool group and school age group (n = 1634)		*χ*^2^	P
	Positive number (n)	Positive rate (%)	Positive number (n)	Positive rate (%)	Positive number (n)	Positive rate (%)		
S. pneumoniae	459	23.48	261	21.48	276	16.89	24.06	0.00*
HRV	82	4.19	77	6.34	46	2.82	21.21	0.00*
RSV	83	4.25	24	1.98	4	0.24	63.93	0.00*
FLUA	7	0.36	20	1.65	74	4.53	76.87	0.00*
C. pneumoniae	7	0.36	5	0.41	63	3.86	84.83	0.00*
M. Pneumoniae	2	0.10	2	0.16	55	3.37	93.32	0.00*
H. influenzae	7	0.36	5	0.41	24	1.47	17.62	0.00*
PIV	19	0.97	5	0.41	4	0.24	8.94	0.01
HPMV	9	0.46	4	0.33	2	0.12	3.28	0.19
HBOV	7	0.36	4	0.33	3	0.18	1.01	0.60
HCOV	7	0.36	1	0.08	2	0.12	3.62	0.16
FLUB	3	0.15	0	0	3	0.18	2.10	0.35
total	692	35.40	408	33.58	556	34.02		

The detection rate of various pathogens was highest in the infant group or preschool group and school age group.

*There were significant differences among the pathogens in different seasons, *P* < 0.05.

### Detection and analysis of respiratory single pathogen infection in children in different seasons

**Figure 3:**
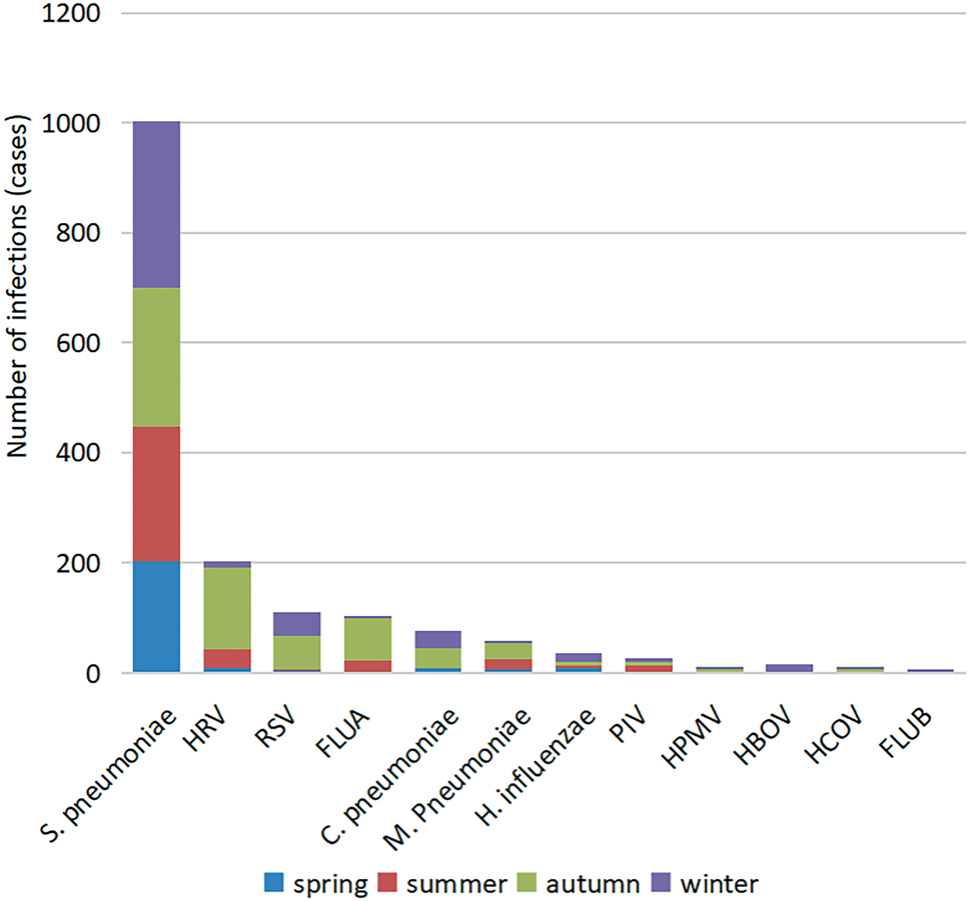
Comparison of the detection rate of single pathogen infection in children with respiratory tract infection in different seasons.

Common pathogens were detected in 4804 children with respiratory tract infection. The positive detection rate was 43.58% (248/569) in spring group and 38.64% (342/885) in summer group. The positive detection rate was 33.73% (628/1862) in autumn and 29.27% (446/1488) in winter. The infection rates of *Streptococcus pneumoniae* and *Haemophilus influenzae* in spring group were higher than those in other three seasons, and the difference was statistically significant (*P* < 0.05); The infection rate of *Mycoplasma pneumoniae* in summer group was higher than that in other three seasons, and the difference was statistically significant (*P* < 0.05); The infection rates of *Rhinovirus*, *Respiratory syncytial virus* and *Influenza A virus* in the autumn group were higher than those in the other three seasons, and the difference was statistically significant (*P* < 0.05); The infection rates of *Chlamydia pneumoniae*, *Boca virus* and *Influenza B virus* in winter group were higher than those in other three seasons, and the difference was statistically significant (*P* < 0.05); There was no significant difference in the positive rates of *Parainfluenza virus*, *Metapneumovirus* and *Coronavirus* infection among different seasonal groups, and there was no statistical significance between different seasonal groups (*P* > 0.05). The results are shown in Table 4 and Fig. 3.

**Table 4:** Comparison of positive rates of respiratory single pathogen infection in children in different seasons.

Pathogen	Spring (n = 569)		Summer (n = 885)		Autumn (n = 1862)		Winter (n = 1488)		*χ*^2^	P
	Positivenumber (n)	Positive Rate (%)	Positive number (n)	Positive rate (%)	Positive number (n)	Positive rate (%)	Positive number (n)	Positive rate (%)		
S. pneumoniae	204	35.85	244	27.57	252	13.53	303	20.36	162.30	0.00*
HRV	10	1.76	34	3.84	148	7.95	12	0.81	115.10	0.00*
RSV	4	0.70	3	0.34	61	3.28	43	2.89	31.66	0.00*
FLUA	0	0	22	2.49	78	4.19	1	0.07	82.19	0.00*
C. pneumoniae	9	1.58	0	0	36	1.93	32	2.15	18.54	0.00*
M. pneumoniae	6	1.05	19	2.15	29	1.56	5	0.34	17.73	0.00*
H. influenzae	9	1.58	5	0.56	6	0.32	17	1.14	30.03	0.00*
PIV	3	0.53	11	1.24	7	0.38	7	0.47	8.39	0.39
HPMV	2	0.35	1	0.11	5	0.27	4	0.27	0.95	0.81
HBOV	1	0.18	0	0	0	0	15	1.01	30.03	0.00*
HCOV	0	0	3	0.34	5	0.27	2	0.13	2.63	0.45
FLUB	0	0	0	0	1	0.05	5	0.34	7.89	0.05
total	248	43.58	342	38.64	628	33.73	446	29.97		

Infection with various respiratory pathogens is higher in autumn and winter than in spring and summer.

*There were significant differences among the pathogens in different seasons, *P* < 0.05.

## Discussion

In this study, 4804 children with respiratory tract infection samples were tested for respiratory tract pathogens by PCR capillary electrophoresis fragment analysis. The results showed that 1656 children were infected with a single pathogen, and the positive detection rate was 34.47%, the pathogens with high detection rate were *Streptococcus pneumoniae, Rhinovirus* and *Respiratory syncytial virus* in order, there were 2085 cases of mixed infection with pathogens, the positive rate was 43.40%. In Zibo area, the rate of detecting a single pathogen was 34.47%, which was slightly lower than the 38.26% found in Kunming area by He Chenglu^4^ and slightly higher than the 32% reported in Tongzhou area of Beijing by Li Hongjun^5^. This rate was significantly lower than the 65.7% reported by Roh EJ^6^ for children in South Korea, indicating that the single pathogen detection rate in Zibo was more in line with the rates in the two Chinese regions and much lower than the overall rate in South Korea. The top three pathogens of respiratory infection in Zibo area were *Streptococcus pneumoniae, Rhinovirus* and *Respiratory syncytial virus*, Chen Jing^7^ reported that among 368 community-acquired pneumonia samples with an average age of 4–5 years old, the pathogens with high detection rate were influenza virus, *Mycoplasma pneumoniae* and *Respiratory syncytial virus*, which was partially consistent with the results of this study, Choe^8^ reported that a total of 7415 positive cases were found in PCR-based respiratory virus testing among children in the United States from 2010 to 2018, among which the top three positive pathogens were *Respiratory syncytial virus*, influenza virus and *Parainfluenza virus*, which was partially consistent with the results of this study. The spread of respiratory pathogens is significantly influenced by location, surroundings, and time of year, and it is important to examine specific regions. This study analyzed the changes of several major respiratory pathogens with age, the results showed that the distribution of common respiratory pathogens in children was closely related to the age of children, and the positive detection rates of infection in infant group (0–1 years old), Toddler group (1–3 years old), preschool group and school age group (3 years old -) were roughly the same. The infection rates of *Streptococcus pneumoniae, Respiratory syncytial virus* and *Parainfluenza virus* in infant group, *Rhinovirus* in Toddler group, *Influenza A virus*, *Chlamydia pneumoniae*, *Mycoplasma pneumoniae* and *Haemophilus influenzae* in preschool group and school age group were higher than those in other age groups, and the difference was statistically significant (*P* < 0.05). The positive detection rate of *Streptococcus pneumoniae* was higher in the infant group, which was consistent with the report of Jin^9^ that *Streptococcus pneumoniae* mostly occurred in infancy, after a survey of serological and antigen testing procedures in 74 children aged 1–9 years with respiratory tract infections in the Gambia, Forgie^10^ found that 77% of the children had bacterial infections and 61% of the bacterial infections were *Streptococcus pneumoniae* infections, similar to the conclusion that the infant group in this study had a higher positive rate of *Streptococcus pneumoniae*. The rates of *Respiratory syncytial virus* and *Parainfluenza virus* were more elevated among infants, but as age increased, there was a noticeable decline. This pattern aligns with findings by Mao^11^, suggesting that infants may be more vulnerable to these viruses due to their underdeveloped respiratory mucosal barrier and local mucosal immunity. The positive detection rate of *Rhinovirus* in Toddler group is relatively high, the positive rate of *Rhinovirus* in the young children group was higher, which was similar to the highest positive rate of *Rhinovirus* found by Furuse^12^ when studying the epidemiology of acute respiratory virus infection in Philippine children, and basically consistent with Li Yan's study^13^, the positive rate also declined with the age of children. The positive rates of *Influenza A virus*, *Chlamydia pneumoniae* and *Mycoplasma pneumoniae* were higher in preschool and school-age children, which was similar to the report of Del^14^ about the high positive rates of *Chlamydia pneumoniae* and *Mycoplasma pneumoniae* in Lima, Peru, which was basically consistent with the research results of Han Lijuan^15^, children older than 3 years old were typically found in kindergartens and primary schools, which served as a hub for local respiratory infection outbreaks. As a result, the pathogens mentioned in the text were mainly prevalent in this age group. The significant occurrence of *Haemophilus influenzae* infection among school-aged children aligns with the observation that this type of infection is more common in children aged 1–14 compared to those under 1 year old, as reported by Gao Xue^16^. This trend is likely attributed to the clustered outbreaks of infection in kindergartens and schools.

The distribution of respiratory pathogens in children is also closely related to seasons, this study analyzed the relationship between several major pathogens of respiratory infections and seasonal changes, the results showed that the positive rates of spring, summer, autumn and winter groups were 43.58%, 38.64%, 33.73% and 29.27%, respectively. *Streptococcus pneumoniae* and *Haemophilus influenzae* in spring group, *Mycoplasma pneumoniae* in summer group, *Rhinovirus*, *Respiratory syncytial virus*, *Influenza A virus* in autumn group; The differences of *Chlamydia pneumoniae*, *Boca virus* and *Influenza B virus* in winter group were statistically significant compared with other groups in different seasons (*P* < 0.05). The spring group showed a higher rate of detecting *Streptococcus pneumoniae* and *Haemophilus influenzae*, aligning with previous findings by Xiao^17^ and Han^18^ indicating increased infection rates of these bacteria in spring and winter compared to summer and autumn. This trend is attributed to the colder weather in spring and winter, the fluctuation between cold and warm conditions, and inadequate parental care leading to children catching colds. In this study, the positive rate of *Mycoplasma pneumoniae* was the highest in summer, Wang^19^ treated 1,700 children with respiratory tract infection in Heyuan City, Guangdong Province, and the results showed that the detection rate of *Mycoplasma pneumoniae* in children with respiratory tract infection in summer was the highest, reaching 31.11%, which was consistent with this study. Li^20^ treated 2520 children with respiratory tract infection in Zunyi area, and the results showed that the detection rate of *Mycoplasma pneumoniae* in children with respiratory tract infection was the highest in winter, reaching 34.15%, which was not consistent with this study, and it was speculated that there were certain differences in the seasons of *Mycoplasma pneumoniae* infection in different regions. The results of this study showed that there was a certain degree of prevalence of *Rhinovirus* and *Influenza A virus* in autumn in Zibo region, which was basically consistent with the high positive rate of *Rhinovirus* and *Influenza A virus* in 292 respiratory tract samples from Shunyi District of Beijing reported by Zhu^21^, the prevalence of *Rhinovirus* in autumn in Zibo was similar to that of *Rhinovirus* in the United Arab Emirates in September reported by Salim^22^ , and the high positive detection rate of *Respiratory syncytial virus* in autumn was inconsistent with the high positive detection rate of *Respiratory syncytial virus* from February to April reported by Zhu^21^, which was consistent with the prevalence rate of *Respiratory syncytial virus* in the United Arab Emirates from August to December reported by Salim S, these results indicate that there are seasonal differences in *Respiratory syncytial virus* infection in different regions. The positive detection rate of *Chlamydia pneumoniae* in this region was higher in winter, which was consistent with the research results reported by Li^23^. The high positive detection rate of *Boca virus* in winter in this region is inconsistent with the high positive detection rate of *Boca virus* in children in Guangzhou in summer reported by Zeng^24^, which may be attributed to the warm and closed environment of winter heating in this region, which provides conditions for the transmission of *Boca virus*. The high positive detection rate of *Influenza B virus* in this region in winter is inconsistent with the report of Zheng^25^ on the high incidence of *Influenza B virus* in children in Shanghai in April. The high incidence season of *Influenza B virus* has regional characteristics, and the prevention and control of the transmission of *Influenza B virus* must be combined with the local epidemiological investigation.

In this study, 4804 samples of children with respiratory tract infection were tested for respiratory tract pathogens by PCR capillary electrophoresis fragment analysis, the results showed that there were 2085 cases of respiratory tract pathogen mixed infection, with a positive rate of 43.40%, the main combinations were *Streptococcus pneumoniae* + *Rhinovirus*, *Streptococcus pneumoniae* + *Haemophilus influenzae* and *Streptococcus pneumoniae* + *Respiratory syncytial virus* accounted for 12.90%, 10.84% and 8.30%, respectively. The reason for the above combination of infections may be that the onset of respiratory tract infection in children is urgent and the children are treated early, at this time, the viral load of most of the infected respiratory tract is low, which is beyond the detection range of PCR capillary electrophoresis fragment analysis, it may be that *Rhinovirus* and *Respiratory syncytial virus* have strong proliferation capacity, and the amount of proliferation is within the detection range of PCR capillary electrophoresis fragment analysis, and thus the above combination of mixed infections occurs. *Streptococcus pneumoniae* and *Haemophilus influenzae* are resident bacteria in the lungs of children, causing respiratory infections when children's immune capacity is weak. Zhu Chanhong^26^ study found that the mixed infection is mainly *Mycoplasma pneumoniae* + *Parainfluenza virus*, the positive detection rate reached 3.28%, the analysis of this study respiratory tract mixed infection and Zhu Chanhong and other studies may be different reasons may be Zhu Chanhong and other studies used immunological technology, because the incidence of respiratory tract infection in children is more urgent, early treatment, IgM detection fell in the window period, the inability to detect pathogen specific IgM antibodies may also be related to the incomplete development of children's immune system and the failure to produce IgM antibodies in a timely and effective manner after pathogen infection. Mixed infection can make the diagnosis and treatment of children's respiratory tract infection more complicated. PCR capillary electrophoresis fragment analysis can be utilized to detect respiratory tract pathogens in children at an early stage of infection, aiding clinicians in promptly determining appropriate treatment plans and guiding clinical medication.

## Conclusion

In summary, the rate of detecting harmful bacteria in children is influenced by their age and the season. To effectively prevent and control infections caused by specific respiratory pathogens, it is important to consider both the age of the child and the season in which the pathogen is most prevalent.

### Supplementary Information

Supplementary Information 1.

Supplementary Information 2.

Supplementary Information 3.

Supplementary Information 4.

Supplementary Information 5.

Supplementary Information 6.

## Data Availability

The study includes all data that was generated or analyzed, which can be found in this document and its supplementary information file. Due to privacy concerns for the large number of children involved, the data sets are not publicly accessible but can be obtained upon request from the authors. However, the availability of the data used in this study is restricted as it was utilized under license.

## Abbreviations

ARI: Acute respiratory infections
*S. pneumoniae*: Streptococcus pneumoniae
HRV: Human rhinovirus
RSV: Respiratory syncytial virus
FLUA: Influenza A virus
*C. pneumoniae*: *Chlamydia pneumoniae*
*M. pneumoniae*: *Mycoplasma pneumoniae*
*H. influenzae*: *Haemophilus* *influenza*
PIV: Parainfluenza virus
HPMV: Human metapneumovirus
HBOV: Human boca virus
HCOV: Human coronavirus
FLUB: Influenza B virus
