# Supplementary material for: Epidemiological characteristics of common respiratory pathogens in children
Source: Sci Rep. 2024 Jul 15;14:16299. doi: 10.1038/s41598-024-65006-3 (PMC11251276; doi:10.1038/s41598-024-65006-3)
Supplement: Supplementary file 2 — Supplementary Information 2. [file 41598_2024_65006_MOESM2_ESM.pdf]

Table 1. Positive rate and proportion of single pathogen infection in respiratory tract

Pathogen	Positive number (n)	Positive rate (%)	Account for (%)
S. pneumoniae	969	20.17	58.51
HRV	205	4.28	12.28
RSV	111	2.31	6.70
FLUA	101	2.10	6.10
C. pneumoniae	75	1.56	4.53
M. Pneumoniae	59	1.23	3.56
H. influenzae	36	0.75	2.17
PIV	28	0.58	1.69
HPMV	16	0.33	0.97
HBOV	13	0.27	0.79
HCOV	10	0.21	0.60
FLUB	6	0.12	0.36
total	1656	34.47	100
