# Supplementary material for: Epidemiological characteristics of common respiratory pathogens in children
Source: Sci Rep. 2024 Jul 15;14:16299. doi: 10.1038/s41598-024-65006-3 (PMC11251276; doi:10.1038/s41598-024-65006-3)
Supplement: Supplementary file 3 — Supplementary Information 3. [file 41598_2024_65006_MOESM3_ESM.pdf]

Table 2. Analysis of mixed infection of respiratory pathogens in children

Pathogen	Positive number (n)	Account for (%)
S. pneumoniae+HRV	269	12.90
S. pneumoniae+H. influenzae	226	10.84
S. pneumoniae+RSV	173	8.30
S. pneumoniae+C. pneumoniae	141	6.76
S. pneumoniae+M. Pneumoniae	121	5.80
S. pneumoniae+FLUB	72	3.45
S. pneumoniae+HRV+H. influenzae	65	3.12
S. pneumoniae+HPMV	58	2.78
S. pneumoniae+H. influenzae+RSV	50	2.40
Other combinations	910	43.65
total	2085	100
