# Supplementary material for: Epidemiological characteristics of common respiratory pathogens in children
Source: Sci Rep. 2024 Jul 15;14:16299. doi: 10.1038/s41598-024-65006-3 (PMC11251276; doi:10.1038/s41598-024-65006-3)
Supplement: Supplementary file 4 — Supplementary Information 4. [file 41598_2024_65006_MOESM4_ESM.pdf]

Table 3. Comparative analysis of respiratory single pathogen infection in children of different age groups

Pathogen	Infant group (n=1955)		Toddler group (n=1215)		Preschool group and school age group (n=1634)		χ^2	*P*
	Positive number (n)	Positive rate (%)	Positive number (n)	Positive rate (%)	Positive number (n)	Positive rate (%)		
S. pneumoniae	459	23.48	261	21.48	276	16.89	24.06	0.00*
HRV	82	4.19	77	6.34	46	2.82	21.21	0.00*
RSV	83	4.25	24	1.98	4	0.24	63.93	0.00*
FLUA	7	0.36	20	1.65	74	4.53	76.87	0.00*
C. pneumoniae	7	0.36	5	0.41	63	3.86	84.83	0.00*
M. Pneumoniae	2	0.10	2	0.16	55	3.37	93.32	0.00*
H. influenzae	7	0.36	5	0.41	24	1.47	17.62	0.00*
PIV	19	0.97	5	0.41	4	0.24	8.94	0.01
HPMV	9	0.46	4	0.33	2	0.12	3.28	0.19
HBOV	7	0.36	4	0.33	3	0.18	1.01	0.60
HCOV	7	0.36	1	0.08	2	0.12	3.62	0.16
FLUB	3	0.15	0	0	3	0.18	2.10	0.35
total	692	35.40	408	33.58	556	34.02		

Note: * There were significant differences amo[illegible]e pathogens in different seasons, $P<0.05$.
