# Supplementary material for: Epidemiological characteristics of common respiratory pathogens in children
Source: Sci Rep. 2024 Jul 15;14:16299. doi: 10.1038/s41598-024-65006-3 (PMC11251276; doi:10.1038/s41598-024-65006-3)
Supplement: Supplementary file 5 — Supplementary Information 5. [file 41598_2024_65006_MOESM5_ESM.pdf]

Table 4. Comparison of positive rates of respiratory single pathogen infection in children in different seasons

Pathogen	Spring (n=569)		Summer (n=885)		Autumn (n=1862)		Winter (n=1488)		$\chi2$	*P*
	Positive number (n)	Positive Rate (%)	Positive number (n)	Positive rate (%)	Positive number (n)	Positive rate (%)	Positive number (n)	Positive rate (%)		
S. pneumoniae	204	35.85	244	27.57	252	13.53	303	20.36	162.30	0.00*
HRV	10	1.76	34	3.84	148	7.95	12	0.81	115.10	0.00*
RSV	4	0.70	3	0.34	61	3.28	43	2.89	31.66	0.00*
FLUA	0	0	22	2.49	78	4.19	1	0.07	82.19	0.00*
C. pneumoniae	9	1.58	0	0	36	1.93	32	2.15	18.54	0.00*
M. pneumoniae	6	1.05	19	2.15	29	1.56	5	0.34	17.73	0.00*
H. influenzae	9	1.58	5	0.56	6	0.32	17	1.14	30.03	0.00*
PIV	3	0.53	11	1.24	7	0.38	7	0.47	8.39	0.39
HPMV	2	0.35	1	0.11	5	0.27	4	0.27	0.95	0.81
HBOV	1	0.18	0	0	0	0	15	1.01	30.03	0.00*
HCOV	0	0	3	0.34	5	0.27	2	0.13	2.63	0.45
FLUB	0	0	0	0	1	0.05	5	0.34	7.89	0.05
total	248	43.58	342	38.64	628	33.73	446	29.97		

Note: * There were significant differences among [illegible] pathogens in different seasons, $P<0.05$.
