# Supplementary material for: Epidemiological characteristics of common respiratory pathogens in children
Source: Sci Rep. 2024 Jul 15;14:16299. doi: 10.1038/s41598-024-65006-3 (PMC11251276; doi:10.1038/s41598-024-65006-3)
Supplement: Supplementary file 6 — Supplementary Information 6. [file 41598_2024_65006_MOESM6_ESM.docx]

S.pneumoniae **Strepto-coccuspneumoniae**, HRV **Human rhinovirus**, RSV **Respiratory Syncytial Virus**, FLUA **Influenza A Virus**, C.pneumoniae **Chlamydia Pneumoniae**, M.pneumoniae **Mycoplasma Pneumoniae**, H.influenzae **Haemophilus influenzae**, PIV **Parainfluenza Virus**, HPMV **Human metapneumovirus**, HBOV **Human boca virus**, HCOV **Human coronavirus**, FLUB **Influenza B virus**.
